# Incidents of Practice

**Published:** 1904-10

**Authors:** W. A. L. Knowles

**Affiliations:** San Francisco.


### INCIDENTS OF PRACTICE.

By W. A. L. Knowles, M. D., D. D. S., San Francisco.

(Written for *The American Journal of Dental Science.*)

Patients are sometimes very positive in regard to certain operations having been performed in the past and though they are mistaken, as proven by books of record and charts, they are sometimes loath to admit their error.

A lady once called at my office, exhibiting a certain filled tooth which she declared had there been operated upon. An examination of the charts, carefully preserved, proved beyond question that the service had been rendered by some other person. The lady insisted that she was correct, that she perfectly remembered the circumstances and stated that she would pit her memory against the best set of books ever kept. The lady was informed that, as no record existed upon the books, if she was correct, she had never paid for the work and must therefore still owe for it. She became angered and left, but after an interval returned to apologize, having recalled the fact that the work in question had been done by another. Her apology was accepted, but future service respectfully declined.

I once had another peculiar experience. A dentist of my acquaintance once sent a lady patient to me to have some carious teeth attended to.

I performed this work and the lady, after getting from me an unfavorable opinion as to the advisability of attempting to save certain molars badly affected by pyorrhoea, returned to her former dentist and placed herself in his hands for treatment of this portion of her work.

This dentist treated these molars much more successfully than I could have done, and when she returned to me to exhibit them, I was more than pleased with the result.

Years of practice have subsequently taught me much in reference to the cure of such cases, an important adjunct to the successful treatment not having been made at all prominent. The lady said to me, "I think that the Doctor wants you to report on this case." I answered, saying that I would be most happy to do so.

The lady had paid a small portion of her bill and informed me that her husband would shortly pay the balance. Circumstances so shaped themselves that I did not have the opportunity to please the gentleman by a flattering report to the Dental Association upon his management of the case.

Some time having elapsed and not receiving payment of my bill, a statement of account was several times sent to the lady's husband and the account was finally placed in the hands of my attorney, who, not succeeding otherwise, was forced to threaten suit.

This threat brought the lady to time, and she called at my office, informing me that she had no intention of paying the balance of her bill as the fillings had all fallen out.

I asked the lady why she had not previously informed me of her dissatisfaction, and stated to her that while it was not unusual to have an occasional filling become dislodged, in my practice of many years I had never had the experience of being informed that all my work had fallen out and I asked the privilege of making an examination of her mouth.

I made an appointment with her, and, in the meantime consulted my books and charted the work which I had performed.

When her appointment hour arrived she seated herself in my chair, and I referred to my diagram and inspected the work as charted.

"Madam," said I, "here is the first and here the second," etc., until I had located all my fillings. They were intact and doing perfect service.

"Well," said she, "that is curious; but look at these open cavities. Where have those fillings gone?"

"Madam," said I, "I can not say. I do not know, because I did not fill them."

"You certainly did," she answered.

"Then you have never been charged for them," I replied, and I once more verified the fillings as charted and compared the charges entered with the items upon the bill, which completely proved my position.

"I beg your pardon," said she. "I do not see how I could have been so mistaken. Send your bill to my husband and he will settle it."

I again rendered the account and, after waiting in vain for several months, made peremptory demand upon the husband, and finally received payment.

Another experience which I had was rather unusual.

A lady, the wife of an army officer, brought to me her young son for examination and consultation.

An estimate was asked for, and the charges appearing satisfactory to her, the lady decided to proceed with the work and made a series of appointments.

The boy was rather delicate, or, at least, was so considered by the mother, he plainly being a "mother's boy," and the lady stipulated that the sittings should be made very short.

There were several unkept appointments, for, unless the sun was brilliantly shining, the lad was never allowed to go out for fear he would catch cold, or that some other dire disaster would befall him.

The work was at last accomplished and the lady directed me to render the account to her husband, saying, "My husband is a medical man and I suppose you will give him a discount."

I replied that it was my custom to extend to medical men a courtesy in the form of a one-third rebate.

I sent the bill to the hotel address given me and, hearing nothing from it, a couple of months later sent another. No response being elicited I sent a messenger to the army officer at his quarters.

Red tape made it extremely difficult to reach the gentleman, but it was at last accomplished. He looked over the bill and said, "Tell the Doctor his bill is too much and if he will cut it in half I will consider it."

When this message was repeated to me, I wrote the gentleman a courteous note, informing him of the contract made upon which I had subsequently made a one-third discount, and stated that the balance I expected to be paid.

His only answer to this was that he would pay half.

I immediately wrote him, that unless my bill was paid within three days, I would commence suit. It was paid next morning.

On an occasion long ago, when the clock attachment for vulcanizing was new, I was called upon by the agent introducing it, who proceeded to explain the manner of operation of the mechanism.

He set the time for one minute, and then, in the course of conversation, directed my attention to some point which required that I should stoop and place my face very close to the clock.

Just as I came near it, the little metal claw was released, and flying back, gouged a small channel in the side of my nose. I assured the agent that his appliance had made quite an impression upon me, and it had.

I once read in a dental journal a description of a method for the removal of a gold crown in an expeditious manner. The process described was to bore a small hole in the top of the crown, insert an excavator, pry a little, and the crown would come off.

Not long afterward I had occasion to remove a gold crown. I bored the hole, inserted the excavator, pried, and the crown came off, but the tooth crown came off, too, inside of it. Perhaps the author of the process forgot to mention that part. I now use a pair of crown slitters and have no further similar experiences.

In regard to cleanliness, or rather uncleanliness of artificial dentures, we have all met with more or less unpleasant experiences.

I have seen instances in which the wings of the denture have been left too high; have first irritated the soft tissues and finally cut their way into them, leaving hanging flops of flesh.

Possibly this embedding of the wings had made the dentures more stable, but it had certainly inflicted much pain upon the wearers.

I have seen a denture in the mouth where the flesh had grown over portions of it, making it necessary to use lance and scissors before the denture could be removed.

We have all met with persons who do not remove the unclean mass of decomposing and fermenting food which clings to dentures.

One of the most peculiar of my experiences was in the mouth of a woman who came from a country town. She was wearing a partial denture of gold clasped to the natural teeth. A clasp on one side had become broken and she had come down to the city to have the plate repaired.

The plate was very foul, and when removed from the mouth was carried at arm's length and placed in a dish of sulphuric acid pickle for boiling. The plate was repaired by the addition of a new clasp and was inserted in the patient's mouth.

"You would better remove it and again place it in position," said I, "to see that it is all right and that you will be able to manage it by yourself, because you must take it out and clean it after each meal."

I used this method to instruct her as to proper cleanliness.

Her answer in her brogue was as follows:

"Shure, I'll not take it out at all. Whin I had the plate first made I wint up to me home in Calistogy and like a fool I tuk thim out and I couldn't put thim back agin, so I had to come clear down to the city agin to have the dintist put thim back. That was sivinteen years ago and I never had thim out of me mouth since."

### METHOD OF MAKING CROWN FOR A DIFFICULT CASE.

By C. Frederick Rogers, D. D. S.,
Conneaut, Ohio.

(Written for *The American Journal of Dental Science.*)

